# Indwelling urinary catheter assembled with lidocaine‐loaded polymeric strand for local sustained alleviation of bladder discomfort

**DOI:** 10.1002/btm2.10218

**Published:** 2021-03-31

**Authors:** Cho Rim Kim, Eun Bi Jang, Seong Hwi Hong, Young Eun Yoon, Beom Kang Huh, Se Na Kim, Min Ji Kim, Hong Sang Moon, Young Bin Choy

**Affiliations:** ^1^ Interdisciplinary Program for Bioengineering, College of Engineering Seoul National University Seoul Republic of Korea; ^2^ Department of Urology, College of Medicine Hanyang University Seoul Republic of Korea; ^3^ Department of Translational Medicine, Graduate School of Biomedical Science & Engineering Hanyang University Seoul Republic of Korea; ^4^ Institute of Medical & Biological Engineering, Medical Research Center Seoul National University Seoul Republic of Korea; ^5^ Department of Biomedical Engineering Seoul National University, College of Medicine Seoul Republic of Korea

**Keywords:** catheter‐related bladder discomfort, indwelling urinary catheters, lidocaine, local drug delivery, sustained drug delivery

## Abstract

Indwelling urinary catheters (IUCs) are used in clinical settings to assist detrusor contraction in hospitalized patients. However, an inserted IUC often causes catheter‐related bladder discomfort. To resolve this, we propose an IUC coupled with local, sustained release of an anesthetic drug, lidocaine. For this, a thin strand composed of poly (lactic‐co‐glycolic acid) and lidocaine was separately prepared as a drug delivery carrier, which was then wound around the surface of the IUC to produce the drug‐delivery IUC. Our results revealed that the drug‐delivery IUC could exert the pain‐relief effects for up to 7 days when placed in the bladder of living rats. Cystometrogram tests indicated that the drug‐delivery IUC could significantly relieve bladder discomfort compared with the IUC without lidocaine. Furthermore, the expression of pain‐related inflammatory markers, such as nerve growth factor, cyclooxygenase‐2, and interleukin‐6 in the biopsied bladder tissues was significantly lower when the drug‐delivery IUC was used. Therefore, we conclude that an IUC simply assembled with a drug‐loaded polymer strand can continuously release lidocaine to allow for the relief of bladder discomfort during the period of IUC insertion.

## 1 INTRODUCTION

Indwelling urinary catheters (IUCs) are placed in the urinary bladder through the urethra to drain urine and relieve urethral retention in hospitalized patients.^1^, ^2^ The IUC is inserted for a short period of 2–4 days, mostly in clinical settings. However, 45%–90% of the patients have reported catheter‐related bladder discomfort (CRBD) (^3^, ^4^). This persistent discomfort is associated with upregulated inflammation of the urethra and bladder after IUC insertion.^5^, ^6^ In addition, patients with IUC experience overactive bladder symptom of involuntary bladder smooth muscle contraction, which is caused by excessive nerve transmission from the bladder detrusor muscle to the muscarinic receptor 3 (^3^, ^7^).

To alleviate CRBD, drugs such as antimuscarinic agents, anesthetics, or anti‐epileptics, are often prescribed as oral bolus and injection. However, these drugs need to be administered in relatively larger doses, which could lead to a risk of systemic toxicity (^3^, ^4^). In this aspect, local administration of an anesthetic drug could be an effective strategy to alleviate CRBD.^8^, ^9^, ^10^ However, even when administered in the form of ointment or gel with a large drug dose, the formulation can be retained at the local target site for only several minutes, resulting in short‐term efficacy.^11^, ^12^ To extend the efficacy period, drugs have been formulated into mucoadhesive particles and liposomes, which can adhere to the mucosal layer in the bladder.^13^, ^14^, ^15^, ^16^, ^17^ A drug‐loaded device has also been proposed, which could be non‐surgically inserted through the urethra in the bladder (^12^, ^18^). These systems could be placed in the bladder for a long time to ensure sustained drug release and prolonged drug efficacy. However, these require separate procedures for drug administration or additional device insertion/retraction, in addition to the insertion of IUC itself.

Therefore, we suggest an IUC capable of sustained release of an anesthetic drug as a single medical device entity with drug delivery functionality. In this study, we separately prepared a thin strand composed of poly (lactic‐co‐glycolic acid) (PLGA) and the anesthetic drug, lidocaine, as a drug delivery carrier. This strand was then assembled with an IUC by wrapping its surface to produce L_PLGA_IUC. As the strand was present only at the surrounding surface of the IUC, the detrusor contraction passage would not be blocked. Therefore, after the insertion of the L_PLGA_IUC via the urethra, lidocaine could be released slowly from the drug‐loaded PLGA strand at the IUC surface, resulting in sustained efficacy in pain relief and anti‐inflammation without additional procedures for drug administration.^12^, ^19^, ^20^, ^21^

In this work, the drug‐loaded PLGA strand was analyzed using scanning electron microscopy (SEM), Fourier transform infrared (FTIR) spectroscopy, and powder X‐ray diffractometry (PXRD). The drug release profiles of the L_PLGA_IUC were assessed under in vitro environments using phosphate‐buffered saline (PBS) (pH 7.4) at 37°C. To examine in vivo efficacy, L_PLGA_IUC was implanted in the normal or cystitis‐induced bladders of living rats. Cystometrogram (CMG) tests were performed to monitor bladder discomfort. In addition, bladder biopsies were performed at the end of the experiments and the tissue samples were assessed for the expressions of nerve growth factor (NGF), cyclooxygenase‐2 (COX‐2), and interleukin‐6 (IL‐6).^22^, ^23^, ^24^

## 2 MATERIALS AND METHODS

### 2.1 Materials

PLGA (lactic acid:glycolic acid = 50:50; MW = 48,000) was obtained from Evonik Industries (Essen, Germany). Dichloromethane (DCM; >99.5%) and *N*,*N*‐dimethylformamide (DMF, >99.5%) were purchased from DaeJung (Siheung, Korea). Acetonitrile (ACN; >99.9%) and lidocaine were obtained from J. T. Bakers (Phillisburg, NJ) and Tokyo Chemical Industry (Tokyo, Japan), respectively. Polethylene catheters (SP‐45 and SP‐61) were obtained from Natsume Seisakusho (Tokyo, Japan). Surgical sutures (4–0 and 5–0 silk) and cotton bandages were purchased from Ailee (Seoul, Korea) and Bandgold (Ansan, Gyeonggi‐do, Korea), respectively. Cyclophosphamide (CYP) was purchased from Sigma‐Aldrich (MO). Anti‐COX‐2 polyclonal antibody (ab 15191), anti‐IL‐6 polyclonal antibody (ab 6672), anti‐NGF polyclonal antibody (ab 6199), and rat IL‐6 ELISA Kit (ab 234570) were obtained from Abcam (MA). DAB kit was purchased from Vector Laboratories (CA). Hematoxylin‐1 and bluing solution were purchased from the Korea Standard (Seongnam, Korea). Revoscript rt premix (25086) was purchased from Invitrogen (Carlsbad, CA). LightCycler ® 480 SYBR Green was purchased from Roche (Basel, Switzerland).

### 2.2 IUC preparation

**FIGURE 1:**
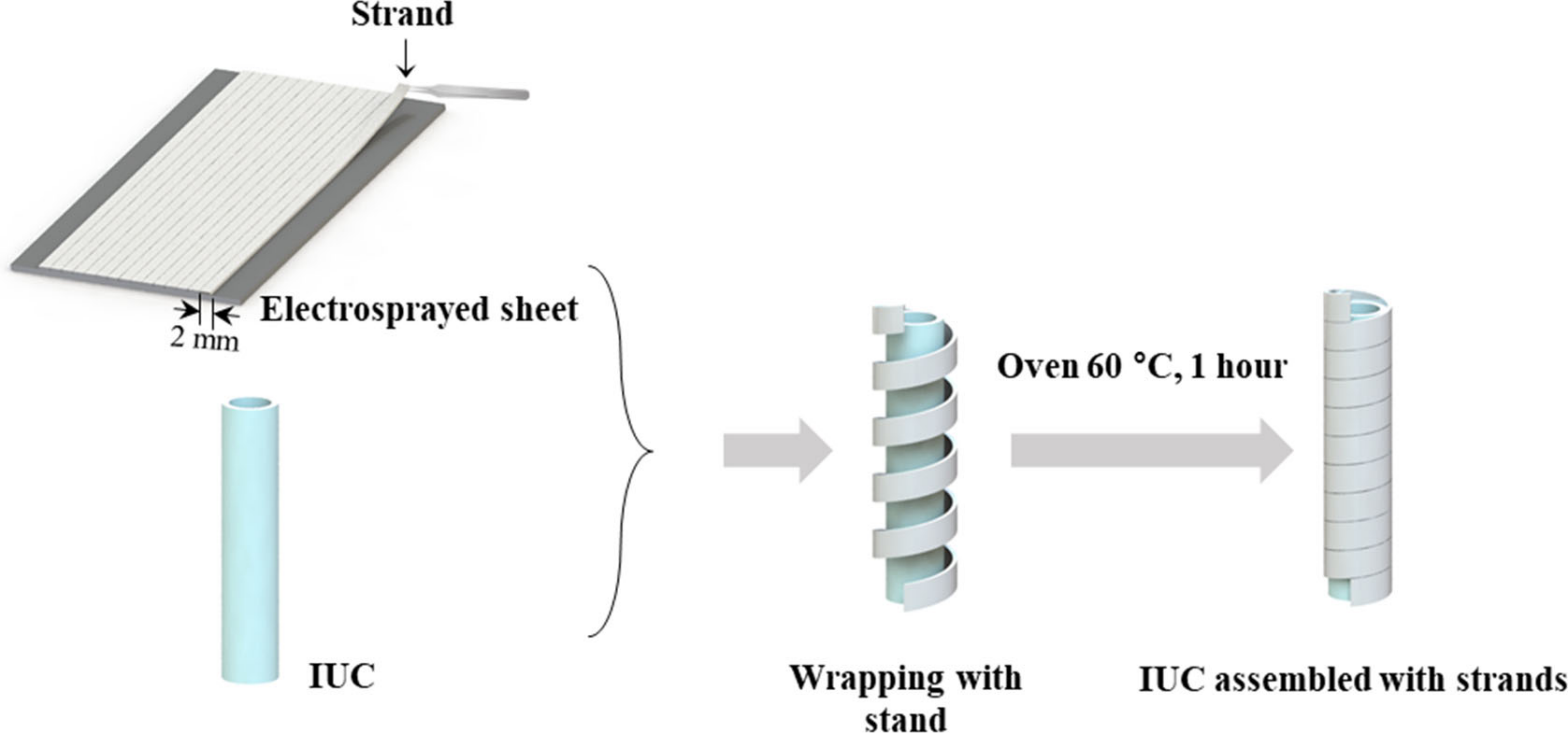
Scheme of the fabrication procedure of strand‐wrapped IUC

To prepare the IUC capable of sustained release of lidocaine (Figure 1), we first fabricated a strand composed of PLGA and lidocaine (L‐PLGA strand). For this, a solution of 4% w/v PLGA and 60% w/w lidocaine was prepared in DCM, which was then electrosprayed (Nano NC, Korea) under the following conditions to produce a sheet: needle size, 24 G; tip‐to‐collector distance, 10 cm; feeding rate, 20 ml/h; and voltage, 20 kV. The sheet was cut into stands, 2 mm in width, which were then used to wrap the surrounding surface of the IUC to produce L_PLGA_IUC. The L_PLGA_IUC was then cured in an oven at 60 °C, slightly higher than the glass transition temperature of PLGA for 1 h to allow for better assembly of the strand with the IUC surface.^25^, ^26^ We also used an IUC wrapped with a strand of PLGA without lidocaine (i.e., PLGA_IUC). For this, we prepared the PLGA strand with a solution of 4% w/v PLGA prepared in DCM without lidocaine, following the same process as described above.

### 2.3 Characterization

The morphologies of the strand and IUC were examined by field emission scanning electron microscopy (FE‐SEM; JSM‐7800F prime, JEOL, Japan). The strands were assessed by FTIR spectroscopy (Tensor 21, Bruker, Germany) at a scanning range of 4000–400 cm^−1^, with a 0.4 cm^−1^ resolution at 298 K, using the KBr disk pellet method. PXRD (SmartLab, Rigaku, Japan) was also performed using Ni‐filtered Cu‐Kα radiation (*λ* = 1.5418 Å) at a tube voltage and current of 3 kW and 30 mA, respectively. For each type of strand, the thickness of at least three samples was measured using a micrometer (Mitutoyo America). To measure the drug loading amount, the L_PLGA_IUC was cut into 1 cm long pieces. Each piece was fully immersed in 1 ml DMF and sonicated for 10 min to completely dissolve PLGA and lidocaine. The lidocaine content in this solution was measured by high‐performance liquid chromatography (HPLC; Agilent 1260 series, Agilent Technologies), equipped with a Diamonsil C18 column (150 × 4.6 mm; Dikma Technologies). The mobile phase consisted of a mixture of 20 mM PBS (pH 2.5) and ACN (80:20, v/v). The absorbance and flow rate were set at 254 nm and 1 ml/min, respectively.

### 2.4 In vitro drug release study

The L_PLGA_IUC, (1 cm in length) was fully immersed in 5 ml of PBS at two distinct pHs of 7.4 and 6.5, respectively, and agitated in a shaking incubator (SI‐600R; Jeio Tech, Seoul, Korea) at 100 rpm at 37°C for 7 days. At predetermined time points, 1 ml of the release medium was collected and replaced with 1 ml of fresh PBS. The collected medium was analyzed by HPLC, as described above.

### 2.5 Animals

**FIGURE 2:**
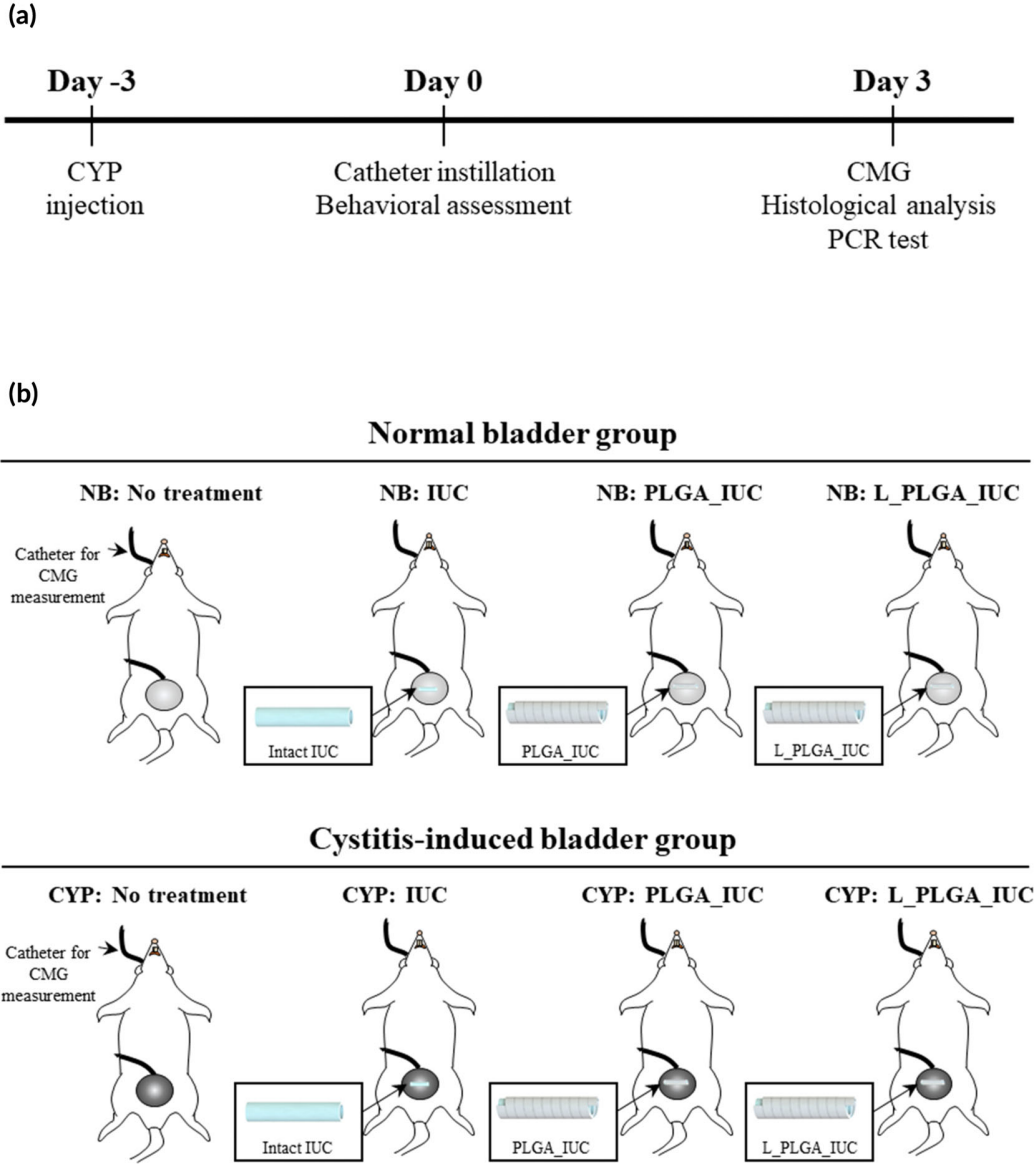
Schematic description of in vivo animal experiments, showing (a) the timeline and (b) animal groups

All experimental procedures were performed using 8‐week‐old female Sprague–Dawley (SD) rats (180 ± 30 g) following institutional guidelines, approved by the Hanyang University Institutional Animal Care and Use Committee (approval number: 2019‐0124). We utilized two different animal models with normal and cystitis‐induced bladders to examine the effect of the anesthetic drug, lidocaine, released from the IUCs prepared in this study. The animal model with a cystitis‐induced bladder was prepared by intraperitoneal injection of 75 mg/kg CYP 3 days prior to IUC insertion.^27^ As shown in Figure 2, we employed eight different groups (*n* = 5 per group):

NB: no treatment; normal bladder without treatment.
NB: IUC; normal bladder implanted with the intact IUC.
NB: PLGA_IUC; normal bladder implanted with the PLGA_IUC.
NB: L_PLGA_IUC; normal bladder implanted with the L_PLGA_IUC.
CYP: no treatment; cystitis‐induced bladder without treatment.
CYP: IUC; Cystitis‐induced bladder implanted with the intact IUC.
CYP: PLGA_IUC; cystitis‐induced bladder implanted with the PLGA_IUC.
CYP: L_PLGA_IUC; cystitis‐induced bladder implanted with the L_PLGA_IUC.

### 2.6 Animal experiment procedures

To insert a differently prepared IUC and locate a polyethylene catheter for CMG analysis, the animal was first anesthetized with respiratory isoflurane. Then, a 3.5 cm‐incision was made in the skin to expose the bladder, and a small incision was made in the bladder dome. Through this incision, the IUC of interest was inserted and placed transversely in the bladder. During this process, we also located a polyethylene catheter for CMG analysis. One end of the polyethylene catheter was located in the bladder, and the incision in the bladder dome was closed with surgical sutures. Then, the other end was passed through the subcutaneous layer tunnel of the left abdomen and exposed outside of the skin near the cervicothoracic joint area. The exposed end of the polyethylene catheter was fixed on the skin using a cotton bandage (Bandgold, Korea), and the skin wound was closed with surgical sutures.

Three days after the implantation, awake CMG analysis was performed on unanesthetized and unrestrained rats in a cage.^28^ For this, the exposed end of the polyethylene catheter was connected to a pressure transducer (PowerLab, ADInstrument, NSW, Australia) and an infusion pump (KD Scientific, Holliston, MA). Normal saline was infused at a rate of 0.15 ml/min and the CMG was plotted versus time for 30 min to 1 h. From the collected CMG data, we assessed the following parameters as indicators of bladder sensitivity: maximum and minimum values of bladder pressure, differences between the two pressures (MAX‐MIN), and intervals between consecutive detrusor contractions.^29^, ^30^

After CMG analysis, the animals were deeply anesthetized with isoflurane, and the bladder and blood samples were promptly extracted. The bladder sample was fixed in 4% buffered formaldehyde for 24–48 h and embedded in paraffin. The blood sample was centrifuged at 3000 rpm for 10 min to obtain the plasma, which was stored at −80°C until further analysis. The animals were then sacrificed following the guidelines of the approved protocol.

### 2.7 Immunohistochemistry (IHC)

For IHC analysis, the paraffin‐embedded bladder was first cut into 3 mm thick sections to prepare the tissue slides. Each section was deparaffinized and rehydrated with alcohol. The slide was treated with antigen retrieval to block endogenous peroxidase activity. To examine the expressions of NGF, COX‐2, and IL‐6, the sections were treated with a corresponding primary antibody overnight at 4°C, followed by incubation with an HRP‐conjugated secondary antibody at room temperature. The slide was then reacted with the DAB kit (Vector Laboratories, Inc.), following the manufacturer's instructions. The slides were also counterstained with hematoxylin‐1 and bluing solution. The slides were observed under an optical microscope (Leica DM 4000B, Leica, Germany) at 200× magnification, and the images were captured using a digital camera (DFC310 FX, Leica, Germany). For each image, the area of stained brown color was quantified in percentage based on the whole tissue area (LAS V4. 1.0; Leica, Germany). For each stained factor, three slides were assessed for each group (*n* = 3).

### 2.8 RNA extraction and quantitative real‐time PCR (qRT‐PCR)

For the bladder tissue samples, total RNA was extracted using TRIzol reagent, which was digested with DNase I and reverse transcribed using Fast Maxime RT premix (25089; Lilif (iNtRON), Seongnam, Korea). Amplification of cDNA was performed with a LightCycler® 480II (Roche, Basel, Switzerland) using the LightCycler® 480 SYBR Green I Master (Roche, Basel, Switzerland). The following gene‐specific primers were employed for cDNA amplification:

NGF‐F: 5′‐TGC ATA GCG TAA TGT CCA TGT TG‐3′
NGF‐R: 5′‐CTG TGT CAA GGG AAT GCT GAA‐3′
COX‐2‐F: 5′‐AAA GCC TCG TCC AGA TGC TA‐3′
COX‐2‐R: 5′‐ATG GTC GCT GTC TTG GTA GG‐3′
IL‐6‐F: 5′‐TGC CTT CTT GGG ACT GAT GT‐3′
IL‐6‐R: 5′‐ACT GGT CTG TTG TGG GTG GT‐3′
GAPDH‐F: 5′‐AGT CTA CTG GCG TCT TCA CCA‐3′
GAPDH‐R: 5′‐AGT TGT CAT GGA TGA CCT TGG‐3′

Relative mRNA expression was calculated by dividing the values of NB: IUC, NB: PLGA_IUC, NB: L_PLGA_IUC or CYP: IUC, CYP: PLGA_IUC, CYP: L_PLGA_IUC by those of NB: No treatment or CYP: No treatment, respectively.

### 2.9 IL‐6 in blood plasma

The level of IL‐6 in the blood plasma sample was measured using a Rat IL‐6 ELISA Kit (ab 234,570; Abcam, USA), following the manufacturer's instructions.

### 2.10 Statistics

Results are presented as the mean ± SE of the mean. Data from CMG, IHC, qRT‐PCR, and ELISA were analyzed by one‐way ANOVA, using GraphPad Prism 7.0 software (GraphPad, CA). Differences were considered statistically significant at *p* < 0.05.

## 3 RESULTS

### 3.1 Strand characterization

**FIGURE 3:**
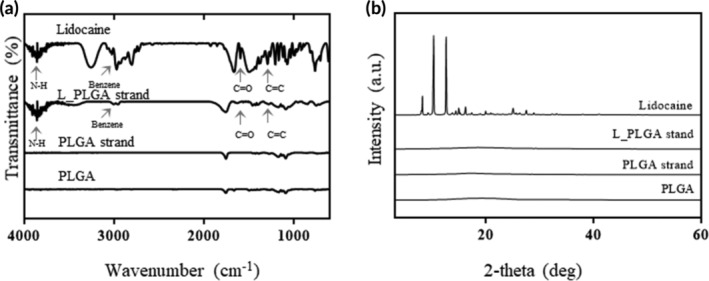
Characterization of the strands. (a) FTIR and (b) PXRD spectra of intact PLGA, lidocaine, PLGA strand, and L_PLGA strand

In this study, we prepared a PLGA‐based strand loaded with lidocaine for sustained drug delivery from the IUC. The strand, fabricated by electrospraying, exhibited a smooth surface regardless of the presence of the drug (Figure S1). As shown in the FTIR spectra in Figure 3a, lidocaine exhibited the characteristic peaks at 3030 cm^−1^, 1630–1690 cm^−1^, 1450–1600 cm^−1^, and 3400 cm^−1^ due to the benzene ring, carbonyl group, C=C, and N—H bonds, respectively.^31^, ^32^, ^33^. These peaks were also observed with the L_PLGA strand, indicating the presence of lidocaine. However, these peaks were not observed with intact PLGA and PLGA strands. As shown in Figure 3b, there were no apparent PXRD peaks seen with the intact PLGA and PLGA strands due to the amorphous structure of PLGA.^34^ Lidocaine showed distinct peaks due to its crystallinity. However, these peaks disappeared with the L_PLGA strand, suggesting that the drug was distributed at the molecular level when the L_PLGA strand was prepared by electrospraying in this study.^35^ The thicknesses of the strands were similar (50.12 ± 2.41 and 51.78 ± 2.1 μm for the PLGA strand and L_PLGA strand, respectively). The L_PLGA strand exhibited a high drug‐loading efficiency of 99.3% (26.78 ± 0.73 μg/mm^2^), as reported with the ones made by electrospraying in previous studies.^36^, ^37^

**FIGURE 4:**
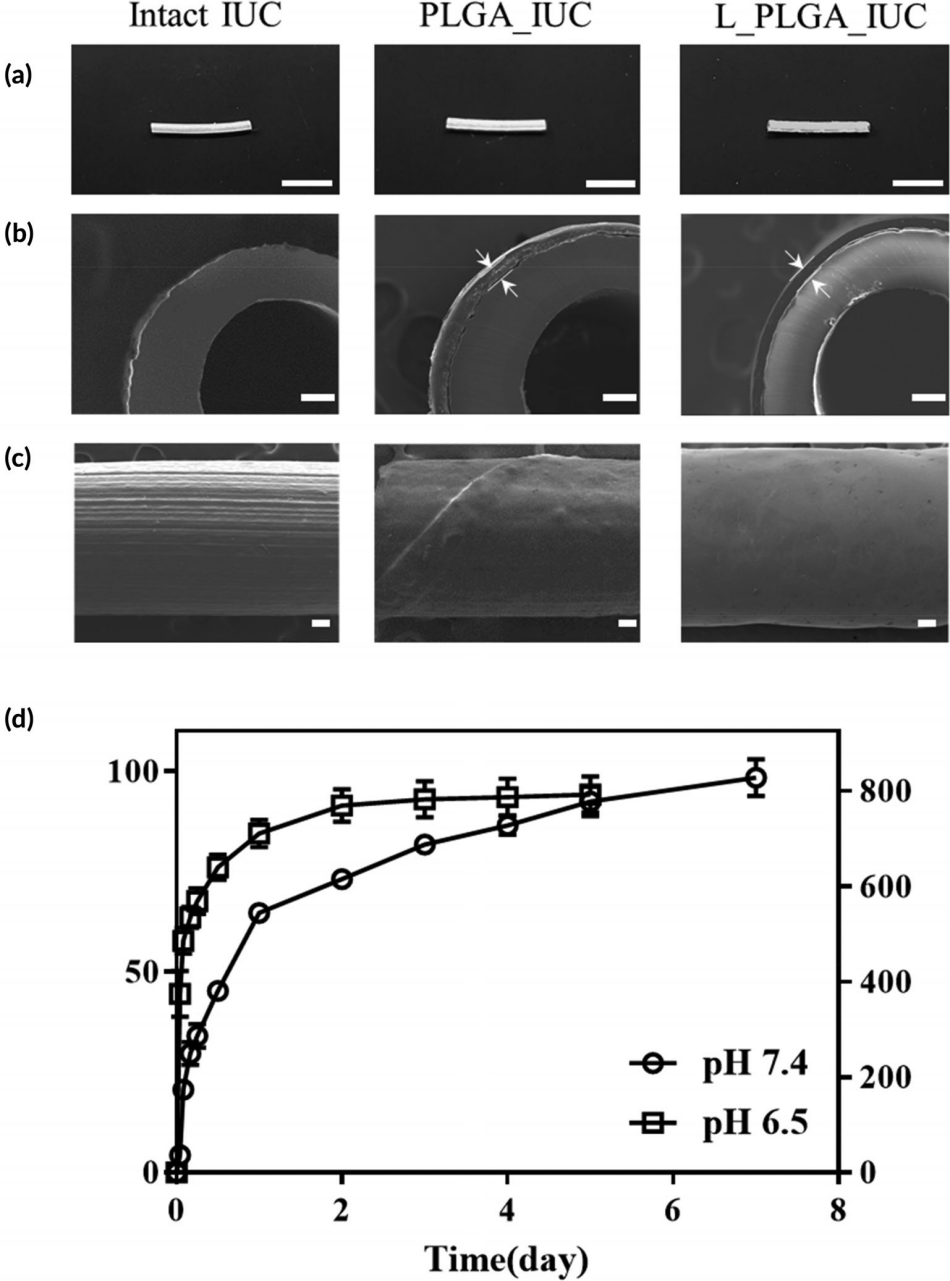
Characterization of the intact and strand‐wrapped IUCs. (a) Optical images of the IUCs cut into 1‐cm long pieces (scale bar = 500 μm). SEM images of (b) cross‐sections and (c) outer‐walls of the IUCs (scale bar = 100 μm). The arrows indicate the thickness of the strands. (d) In vitro lidocaine release profiles of the L_PLGA_IUC at pH 7.4 and 6.5. Each value represents the mean ± SE

The IUC was then wrapped with the strands prepared to produce the PLGA_IUC or L_PLGA_IUC, as shown in Figure 4a. The SEM images confirmed seamless adherence of the strand to the IUC surface without an apparent change in strand thickness and surface morphology (Figure 4b,c). The strand did not overlap after winding, thus allowing for an accurate and reproducible drug loading per cm of IUC, which was measured to be 841 ± 23 μg/cm for the L_PLGA_IUC. As shown in Figure 4d, L_PLGA_IUC exhibited sustained drug release for more than 5 days at both pHs of 7.4 and 6.5. At pH 7.4, after an initial release of about 64.7 ± 0.8% (543.8 ± 6.5 μg/cm) during the first day, drug was released slowly at a rate of 6.8 ± 0.6%/day (65.8 ± 5.0 μg/cm/day). The drug release was faster at pH 6.5, the condition of which might mimic the biological fluid in the bladder more closely.^38^ Probably due to a higher solubility of lidocaine in acidic medium,^39^ the initial release of lidocaine was higher (669.3 ± 22.9 μg/cm) compared to that at pH 7.4, after which the drug was released at a rate of 0.6 ± 0.1% (5.0 ± 1.2 μg/cm/day) until 5 days. The release rates of lidocaine at both pHs were close to the effective dose needed for local pain relief.^40^

### 3.2 CMG analysis results

To examine the efficacy of sustained‐release lidocaine, we assessed the CMG profile of the animals with a normal or cystitis‐induced bladder implanted with different types of IUCs. Animals with a cystitis‐induced bladder represent a bladder pain syndrome involving an overactive bladder, urinary urgency, and high inflammation in the bladder.^41^ CMG analysis is a diagnostic procedure that can assess bladder function,^42^ a higher sensitivity of the bladder shows an increase in bladder pressure (i.e., the maximum and minimum pressures and the difference between the two) and a decrease in the interval between consecutive detrusor contractions.

**FIGURE 5:**
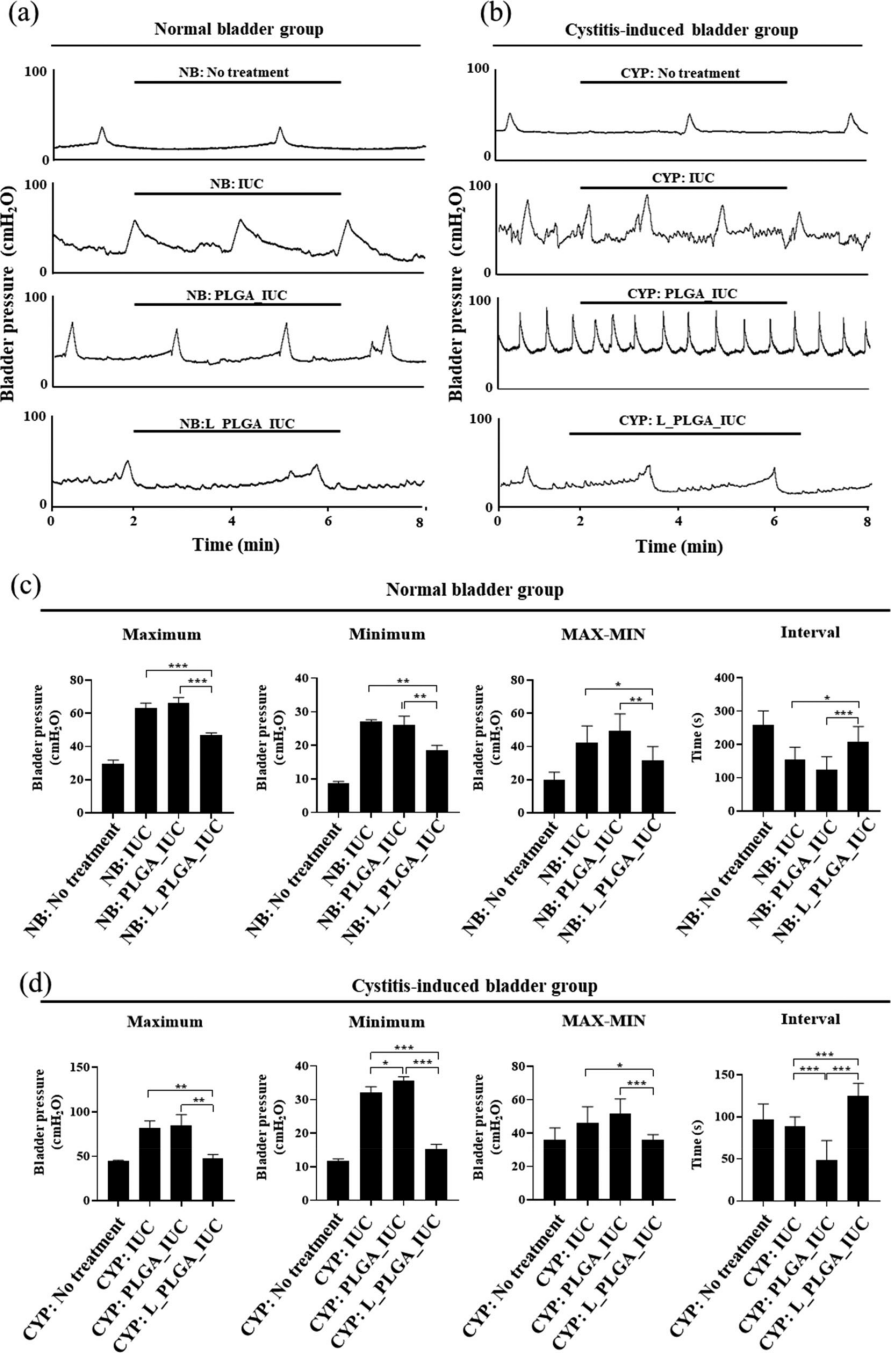
In vivo CMG test results 3 days after IUC insertion in normal and cystitis‐induced bladders. (a and b) Representative CMG curves and (c and d) their quantitative results of maximum pressure, minimum pressure, MAX‐MIN pressure and interval between consecutive detrusor contractions. Each value represents the mean ± SE (*p < 0.05, **p < 0.01, ***p < 0.001)

Figure 5a shows the CMG results in a normal bladder animal model. Reproducible micturition cycles were observed in all groups. However, it was observed that in the rats implanted with the IUCs (i.e., NB: IUC, NB: PLGA_IUC and NB: L_PLGA_IUC), the average bladder pressure increased and the average interval of detrusor contractions decreased compared with NB: No treatment. This suggests that the insertion of an IUC itself increased bladder sensitivity. However, these values were not very different between NB: IUC and NB: PLGA_IUC, indicating that the PLGA strand around the IUC did not affect bladder sensitivity in the normal bladder. It should be noted that as the capacity of sustained lidocaine release in NB: L_PLGA_IUC improved, the bladder pressure decreased and the interval of detrusor contractions became longer. Considering that the CMG analysis was performed 3 days after IUC insertion, the results indicated that the drug was still present and effective in the bladder due to a sustained drug release property of the L_PLGA_IUC. As shown in Figure 4d (Figure S2), the efficacy of the L_PLGA_IUC was also apparent when tested in the cystitis‐induced bladder. Decrease in bladder pressure and increase in interval of detrusor contractions were observed in CYP: L_PLGA_IUC, which were significantly different from those in CYP: IUC and CYP: PLGA_IUC, respectively.

### 3.3 NGF, COX‐2, and IL‐6 expression

**FIGURE 6:**
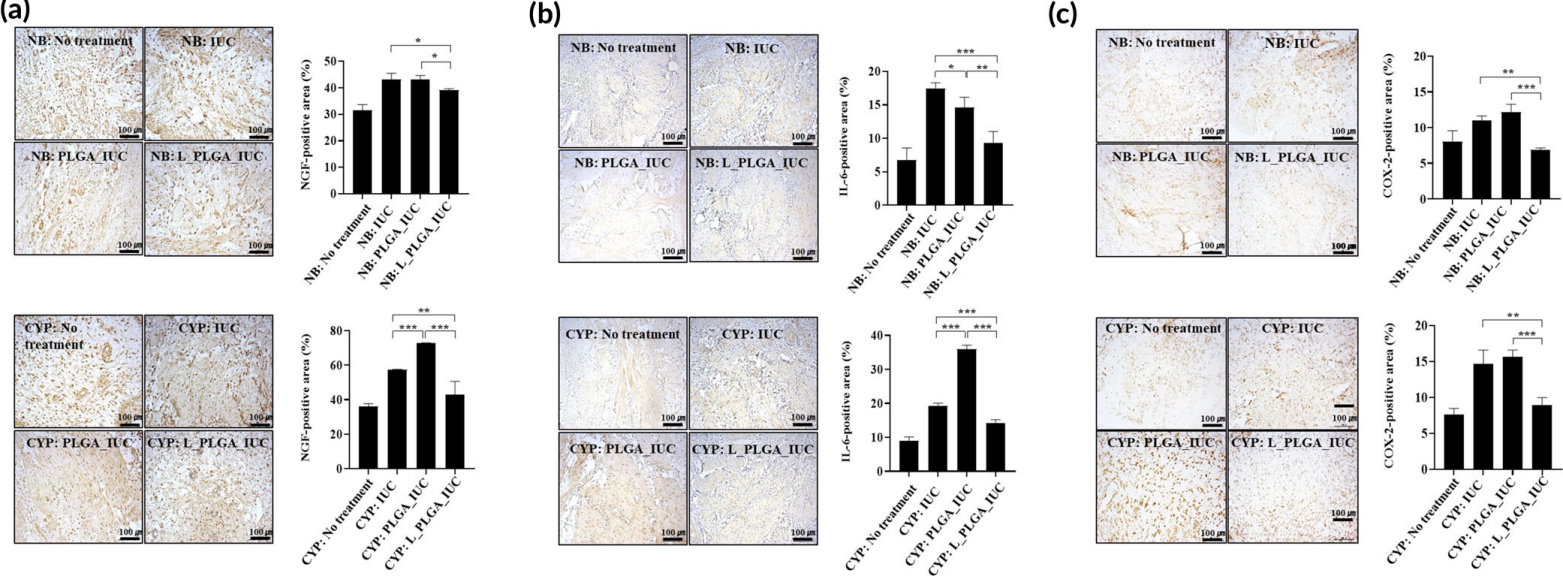
IHC analysis of the bladder tissues biopsied 3 days after IUC insertion. The factors evaluated were (a) NGF, (b) COX‐2, and (c) IL‐6. Scale bars = 100 μm. Each value represents the mean ± SE (*p < 0.05, **p < 0.01, ***p < 0.001)

**FIGURE 7:**
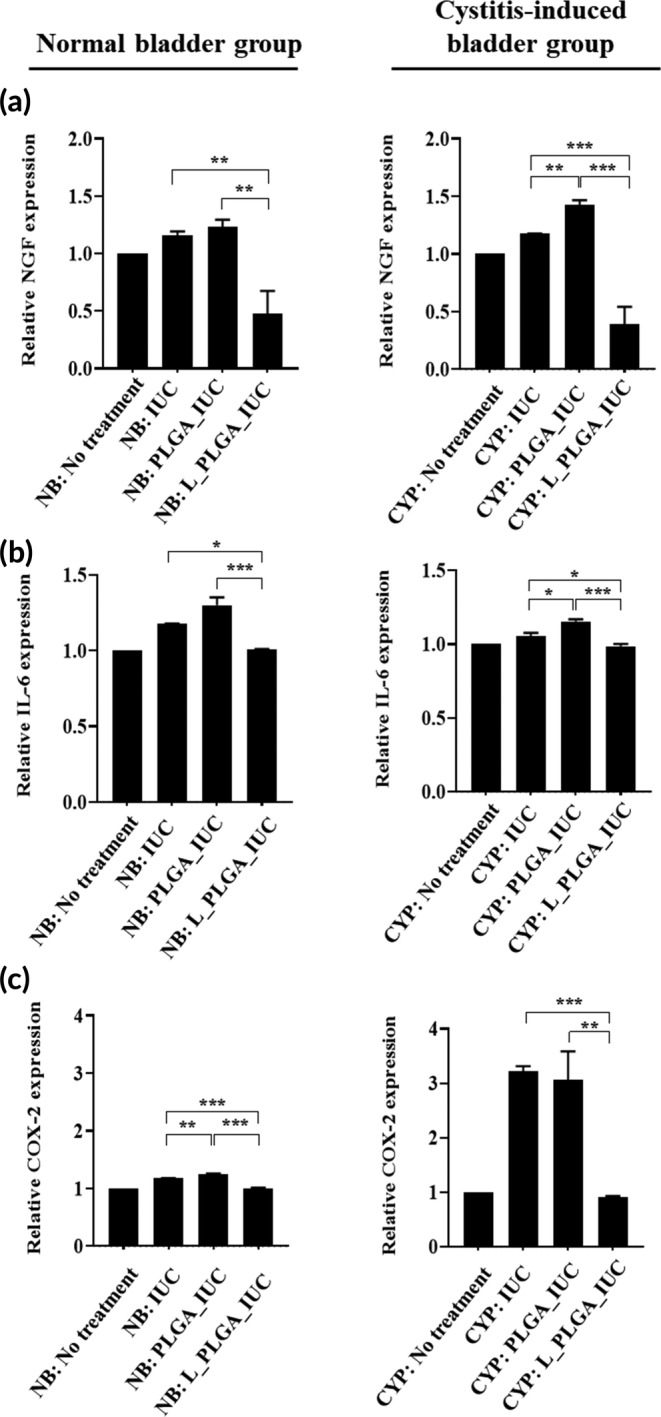
mRNA expression of (a) NGF, (b) COX‐2, and (c) IL‐6 in the bladder tissues biopsied 3 days after IUC insertion. Each value represents the mean ± SE (*p < 0.05, **p < 0.01, ***p < 0.001)

**FIGURE 8:**
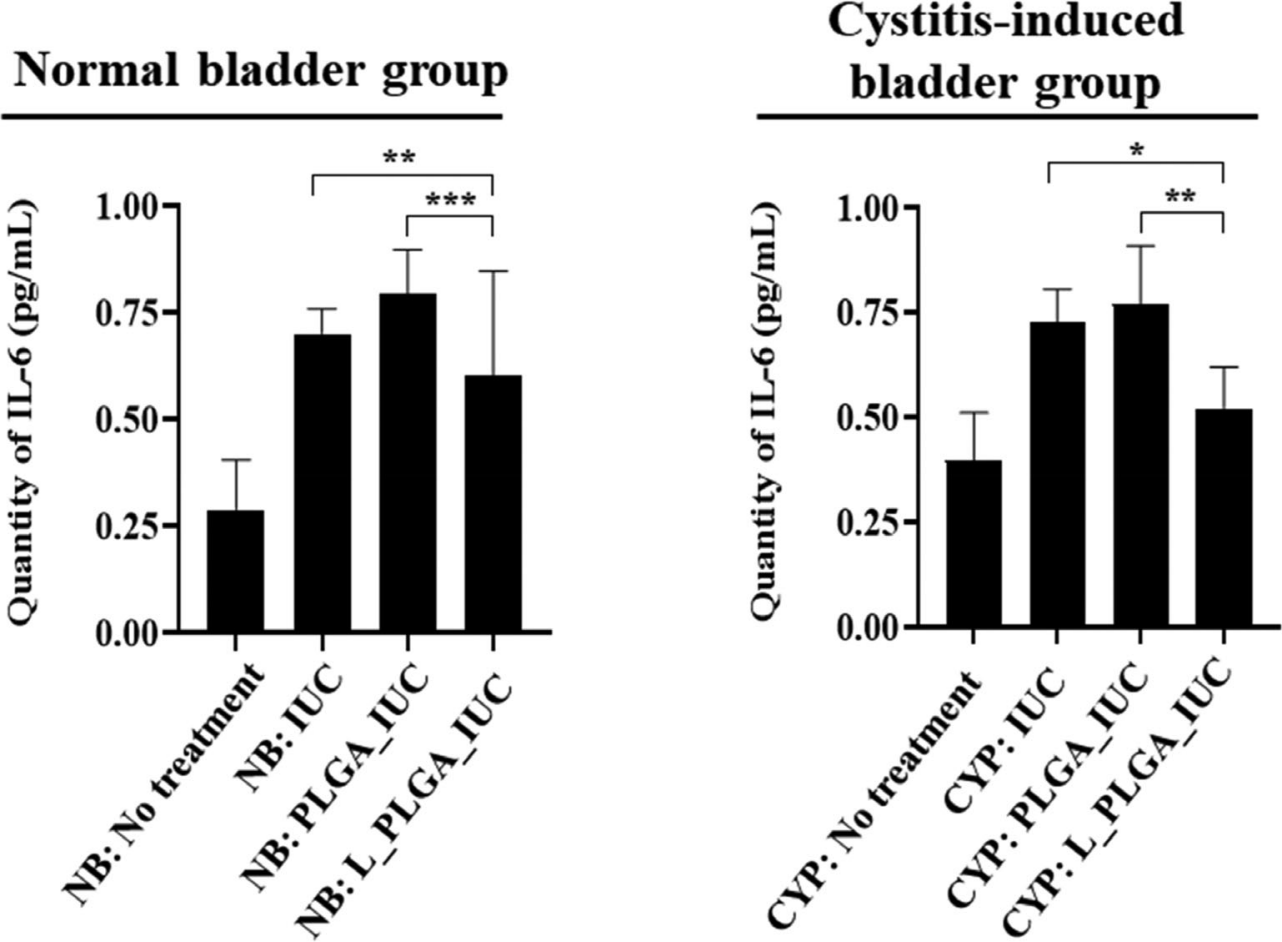
IL‐6 levels in blood plasma obtained 3 days after IUC insertion. Each value represents the mean ± SE. (*p < 0.05, **p < 0.01, ***p < 0.001)

We also examined the expression of NGF, COX‐2, and IL‐6 in the bladder tissues biopsied 3‐day post implantation. NGF is known to be highly relevant to pain and sensitivity.^43^, ^44^ COX‐2 is responsible for pain and inflammation.^45^, ^46^ IL‐6 is known to act as a pro‐inflammatory cytokine.^47^ As shown in Figure 6, the animals treated with L_PLGA_IUC exhibited a statistically significantly lower expression of all factors of NGF, COX‐2, and IL‐6 than the control groups in each bladder model, which was further confirmed by the expression of the corresponding mRNAs (Figure 7). The results suggested that sustained exposure of lidocaine from the L_PLGA_IUC reduced the expression of pain‐ and inflammation‐related factors in the bladder tissue, thereby reducing pain and lowering sensitivity (Figure 5). This appeared to also influence the systemic level of IL‐6, a pro‐inflammatory cytokine, and the level of IL‐6 in blood plasma was significantly lower in NB: L_PLGA_IUC and CYP: L_PLGA_IUC, when compared with the groups implanted with the IUCs without lidocaine in each bladder model (Figure 8).

## 4 DISCUSSION

IUC insertion is commonly performed to relieve urinary retention and monitor urinary output in clinical settings. However, this often leads to CRBD, causing irritation or even a burning sensation in the urethra and bladder.^9^ To treat this, additional drug therapy is often performed, where drugs are prescribed for systemic or local effects.^9^ However, the period of drug efficacy is short, and thus more frequent drug administrations or a higher drug dose may be needed, which can cause adverse side effects. In this aspect, an IUC coupled with local, sustained delivery of an anesthetic drug can be beneficial as the discomfort can be relieved during the period of IUC insertion without any additional procedures.

In previous studies, IUC has been mostly dip‐coated or spray‐coated with a solution of drug or a mixture of drug and polymer, which allows local and sustained drug release.^48^, ^49^, ^50^, ^51^, ^52^, ^53^ Thus, the material of the IUC itself and/or the coated polymer serves as the drug diffusion mediator to allow for sustained drug release. However, when using these conventional coating methods, it may not be easy to control the homogeneity or uniformity of the coating layer throughout the IUC. Moreover, the direct deposition of the toxic coating solution (organic solvent)^50^, ^54^, ^55^, ^56^, ^57^ may result in a greater tendency of the solvent to be trapped in the hydrophobic silicone surface of an IUC.^58^ In case of the dip‐coating method, both the inner and outer walls of an IUC are coated together. This results in doubling of the drug dose, while the drug in the inner wall may not be needed to relieve the CBRD, which occurs in the tissues actually in contact with the outer wall of the IUC. The coating in the inner wall may also change the fluid resistance through the IUC^49^, ^50^, ^52^, ^53^ thereby affecting the drainage of urine.

Therefore, we developed a combined unit of a drug‐delivery IUC, consisting of an IUC assembled with a thin strand, prepared separately with lidocaine‐loaded PLGA (Figure 4). Our results revealed that the drug‐loaded strand could attach well around the outer surface of the IUC, showing a fairly uniform drug distribution (841 ± 23 μg/cm), and thus, the drug was released only from the IUC surface in a sustained manner for more than 5 days. For inpatients in clinical settings, an IUC is inserted in the urethra for about 4 days,^59^ during which the L_PLGA_IUC can be envisioned to relieve local discomfort with a drug release rate of more than 5.0 ± 1.2 μg/cm/day (Figure 4d).^40^ The dose and period of drug release can be easily modulated as needed by varying the strand composition, without altering the major characteristics of the IUC.^57^, ^60^ The drug release profile can be further modulated by employing PLGA of different ratios between lactic and glycolic acids, molecular weights or polymer end groups.^61^, ^62^, ^63^

Our findings revealed that the L_PLGA_IUC could indeed relieve bladder discomfort induced by IUC insertion in the animal models. In bladders with two different sensitivities, the pressure was lowered and the interval between consecutive detrusor contractions was longer compared with the IUC without lidocaine (Figure 5). When assessed during the whole period of 7 days until drug release was completed, the detrusor contraction interval was observed to be significantly lower than that of the IUC without lidocaine at all the time points examined (Figure S2). The maximum fluid capacity in the rat bladder is known to be about 1 ml, where about 0.26 ml of new fluid is filled and urinated every 20 min.^64^, ^65^ Thus, a total volume of the fluid available in the rat bladder for a day could be calculated to be about 20 ml. As the L_PLGA_IUC herein would release more than 5.0 μg lidocaine per day (Figure 4d), the drug concentration in the bladder was expected to be maintained higher than 250 ng/ml. This concentration is higher than that known to be effective for a local pain relief in tissues.^40^

The pain relief and anti‐inflammatory effect of lidocaine was also apparent in the bladder tissues,^23^, ^66^ and L_PLGA_IUC exhibited significantly lower expression of NGF, COX‐2, and IL‐6 compared with the IUCs without lidocaine (Figures 6 and 7).^22^, ^23^, ^67^ However, with the cystitis‐induced bladder, overall inflammation was higher with the PLGA_IUC when compared with that of the intact IUC (Figures 6 and 7), which also appeared to affect the bladder sensitivity (Figure 5d). This could be ascribed to PLGA degradation, producing acidic by‐products at the local site.^68^, ^69^ In this sense, the biocompatible polymers with a slower degradation or non‐degradation could be envisioned as alternative drug‐carrier materials for the IUC.^70^, ^71^ In an aspect of clinical applications, the L_PLGA_IUC may need to be inserted through the urinary tract without detachment of the drug‐loaded strand. Considering this, after assembly, the L_PLGA_IUC herein was cured at above the glass transition temperature of PLGA, as done in our previous studies, where the assembled device could be well retained in structure and drug release profile.^26^, ^72^, ^73^

## 5 CONCLUSION

We propose an IUC coupled with local, sustained release of an anesthetic drug, lidocaine, for the purpose of CRBD alleviation in the bladder. This drug‐delivery IUC can be prepared by a simple assembly process, where the surface of an IUC is wrapped with a strand made of a biocompatible polymer PLGA and lidocaine. In this way, the drug‐delivery IUC can still retain its own function of aiding detrusor contraction while the drug can be released in a sustained manner locally at the site of IUC insertion. Our results showed that the drug‐delivery IUC could release lidocaine over a period of time, as generally required during IUC insertion in clinical settings. When tested in both normal and cystitis‐induced bladders in vivo, the drug‐delivery IUC showed significant effects in alleviating bladder sensitivity, pain, and inflammation. Based on the obtained results, it can be concluded that the developed drug‐delivery IUC is a promising device to help urine drainage with a minimized complication of CRBD.

## CONFLICT OF INTEREST

The authors declare that they have no known competing financial interests or personal relationships that could have appeared to influence the work reported in this paper.

## Supporting information


**Figure S1** SEM images of the surface of (a) PLGA strand and (b) L_PLGA strand (scale bars = 100 μm).
**Figure S2**. In vivo CMG test results at 1, 3, 5, and 7 days after the insertion of the intact catheter and L_PLGA_IUC in normal bladders. During the entire testing period, the L_PLGA_IUC group showed a significantly longer interval of consecutive detrusor contractions compared with the intact catheter group (*p < 0.033, **p < 0.002, ***p < 0.001, ****p < 0.0001).

## Data Availability

Data available on request from the authors
