# Supplementary material for: Indwelling urinary catheter assembled with lidocaine‐loaded polymeric strand for local sustained alleviation of bladder discomfort
Source: Bioeng Transl Med. 2021 Mar 31;6(2):e10218. doi: 10.1002/btm2.10218 (PMC8126825; doi:10.1002/btm2.10218)
Supplement: Supplementary file 1 — Figure S1 SEM images of the surface of (a) PLGA strand and (b) L_PLGA strand (scale bars = 100 μm). Figure S2. In vivo CMG test results at 1, 3, 5, and 7 days after the insertion of the intact catheter and L_PLGA_IUC in normal bladders. During the entire testing period, the L_PLGA_IUC group showed a significantly longer interval of consecutive detrusor contractions compared with the intact catheter group (*p < 0.033, **p < 0.002, ***p < 0.001, ****p < 0.0001). [file BTM2-6-e10218-s001.docx]

*
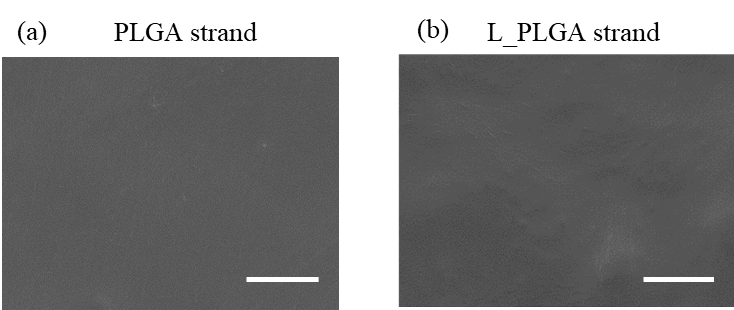
*

Fig. S1. SEM images of the surface of (a) PLGA strand and (b) L_PLGA strand (scale bars = 100 μm).


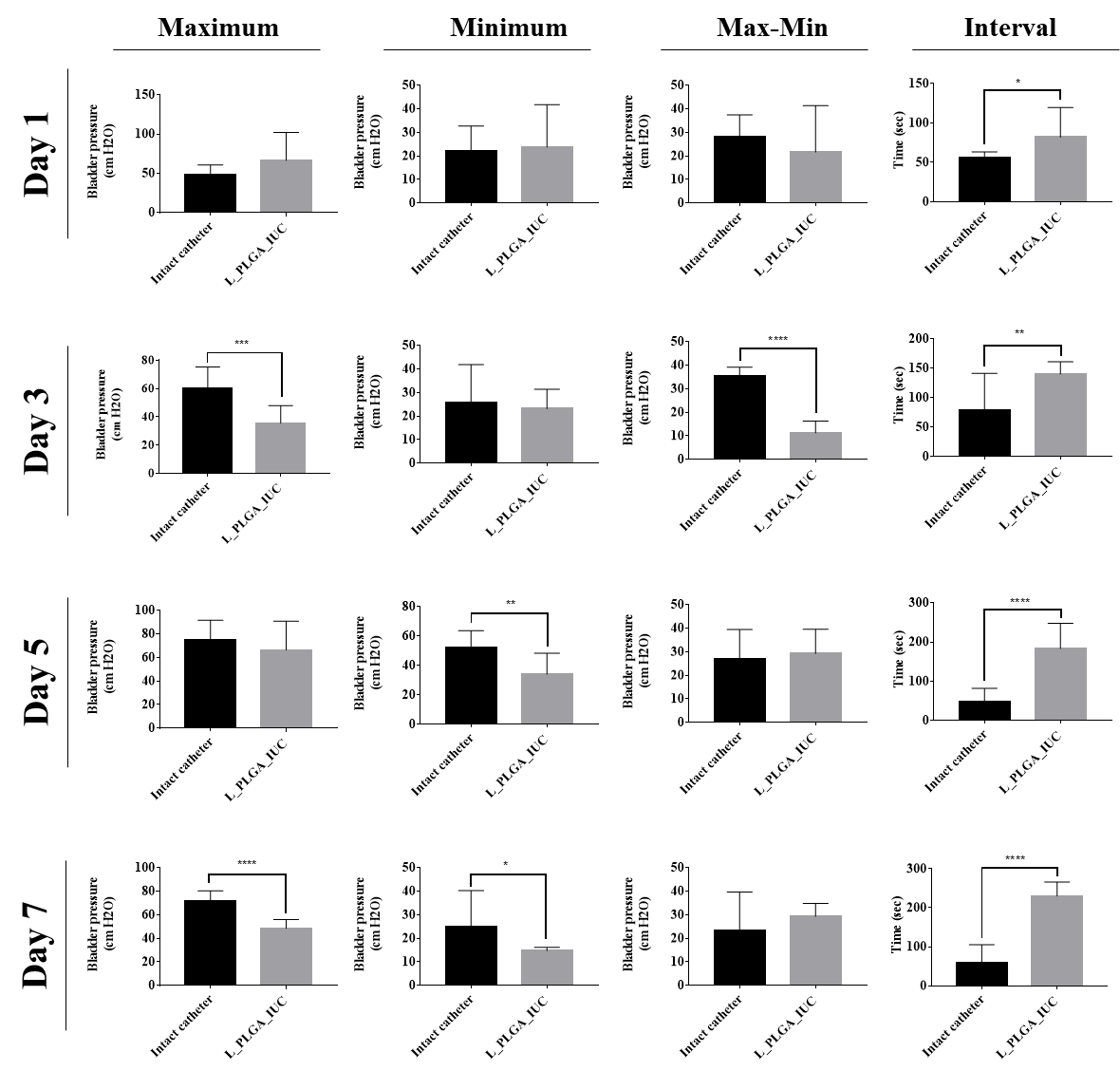


Fig S2. *In vivo* CMG test results at 1, 3, 5, and 7 days after the insertion of the intact catheter and L_PLGA_IUC in normal bladders. During the entire testing period, the L_PLGA_IUC group showed a significantly longer interval of consecutive detrusor contractions compared with the intact catheter group. (*p < 0.033, **p < 0.002, ***p < 0.001, ****p < 0.0001).
